# Supplementary material for: Mechanisms for collective inversion-symmetry breaking in dabconium perovskite ferroelectrics
Source: J Mater Chem C Mater. 2021 Feb 16;9(8):2706–11. doi: 10.1039/d1tc00619c (PMC8905487; doi:10.1039/d1tc00619c)
Supplement: TC-009-D1TC00619C-s001 [file TC-009-D1TC00619C-s001.pdf]

Electronic Supplementary Material (ESI) for Journal of Materials Chemistry C.
This journal is © The Royal Society of Chemistry 2021

Mechanisms for collective inversion-symmetry breaking in dabconium perovskite ferroelectrics:

SUPPLEMENTARY INFORMATION

Dominic J. W. Allen,[a] Nicholas C. Bristowe,[b] Andrew L. Goodwin,[a,*] and Hamish H.-M. Yeung[a,c,*]

[a]Department of Chemistry, University of Oxford, Inorganic Chemistry Laboratory, South Parks Road, Oxford OX1 3QR, U.K.

[b]Department of Physics, Durham University, South Road, Durham DH1 3LE, U.K.

[c]School of Chemistry, University of Birmingham, Edgbaston, Birmingham B15 2TT

*To whom correspondence should be addressed;

E-mail: andrew.goodwin@chem.ox.ac.uk or h.yeung@bham.ac.uk

Contents

1 Monte Carlo simulations **3**

2 Dipole–dipole and strain calculations **6**

3 Density functional theory calculations **7**

4 Hydrogen bond strengths in methyldabconium perovskites **14**

5 Symmetry implications of (anti)ferroelastic distortions **16**

6 References **17**

1 Monte Carlo simulations

Methodology

Metropolis Monte Carlo (MC) simulations were carried out using custom code based on that employed in Ref. S1 The fundamental degrees of freedom in all simulations were the MDABCO orientations, which were treated as classical unit spin vectors $\mathbf{S}_i$ arranged on a simple cubic lattice. The $\mathbf{S}_i$ were nearly always treated as Potts states; *i.e.* only a fixed number of possible orientations were possible for a given model—usually the eight possible $\langle 111 \rangle$ vectors. We discuss below the implications of using Heisenberg degrees of freedom with strong single-ion anisotropy. In general we used simulation boxes containing an $8 \times 8 \times 8$ supercell of the primitive aristotypic cell (*i.e.* 512 'spins') and periodic boundary conditions were applied. Simulations were repeated in independent multiples of five. Equilibration times were estimated based on the number of MC steps required for the autocorrelation function to vanish within a specified (small) limit. For a given MC temperature point, each simulation was allowed to run for ten times as many moves as the corresponding equilibration time, and thermodynamic values were averaged over five successive collection runs, each spaced by this same number of MC steps. This means that the data points shown in Fig. 4(a) of the main text, for example, were each obtained as the average over 25 independent MC configurations.

MC energies were calculated using various combinations of the following various terms described in the text:

$$E_{\text{HB}} = H \sum_{j \in \{\text{X}\}} (n_j - 1)^2, \tag{1}$$

$$E_{\text{dip}} = D \sum_{i \neq j} \frac{\mathbf{S}_i \cdot \mathbf{S}_j - 3(\mathbf{S}_i \cdot \hat{\mathbf{r}}_{ij})(\mathbf{S}_j \cdot \hat{\mathbf{r}}_{ij})}{(r_{ij}/a)^3}, \tag{2}$$

$$E_{\text{strain}} = -J \sum_{i,j} (\mathbf{S}_i \cdot \mathbf{S}_j)^2, \tag{3}$$

$$E_{\text{aniso}} = \Theta \sum_i (S_{ix}^4 + S_{iy}^4 + S_{iz}^4). \tag{4}$$

The dipolar term was calculated using Ewald summation, giving a maximum relative error of 3.8×10^{-5}. Our implementation follows that of Ref. S1, which in turn is based on the implementations in Refs. S2–4.

Two types of MC simulations were carried out. On the one hand, we sought sometimes to establish the ground state for a given interaction model. In such cases we used a simulated annealing approach as appropriate. On the other hand, we wished to determine the temperature dependence of other interaction

models—specifically identifying the existence and nature of order/disorder phase transitions. In such cases we started our MC simulations at a temperature three times that of the dominant interaction term energy, and cooled at a relative rate of either 5% or 3% between successive MC temperature steps. A summary of the various MC simulations and their output is given in Table S1.

Effect of single-ion anisotropy

We make the point in the main text that allowing some deviation from $\langle 111\rangle$ orientations in MC simulations of the key dipole–dipole + strain model preserves the paraelectric/ferroelectric phase transition but lowers the corresponding transition temperature. In support of this statement we report here the results of a MC simulation with anisotropic Heisenberg degrees of freedom. Note that the term E_{aniso} [Eq. (4)] is minimised for $\mathbf{S}_i \in \frac{1}{\sqrt{3}}\langle 111\rangle$, and so Θ captures the energy scale associated with deviations away from $\langle 111\rangle$. The results of this anisotropic Heisenberg MC simulation are shown in Fig. S1 for two values $\Theta = 5J, 10J$, where they are compared against the Potts-model results given in Fig. 4(a) of the main text.

Degrees of freedom	MC Energy	Type	Result
8-state Potts $\mathbf{S}_i \in \frac{1}{\sqrt{3}}\langle 111\rangle$	$E_{\text{MC}} = E_{\text{HB}}$	G. S.	Fig. 2(a)
8-state Potts $\mathbf{S}_i \in \frac{1}{\sqrt{3}}\langle 111\rangle$	$E_{\text{MC}} = E_{\text{dip}}$	G. S.	Fig. 2(b)
8-state Potts $\mathbf{S}_i \in \frac{1}{\sqrt{3}}\langle 111\rangle$	$E_{\text{MC}} = E_{\text{dip}} + E_{\text{strain}}; D = J$	T. D.	Fig. 4(a)
12-state Potts $\mathbf{S}_i \in \frac{1}{\sqrt{2}}\langle 110\rangle$	$E_{\text{MC}} = E_{\text{dip}} + E_{\text{strain}}; D = J$	T. D.	Fig. 4(a)
6-state Potts $\mathbf{S}_i \in \langle 100\rangle$	$E_{\text{MC}} = E_{\text{dip}} + E_{\text{strain}}; D = J$	T. D.	Fig. 4(a)
Anisotropic Heisenberg	$E_{\text{MC}} = E_{\text{dip}} + E_{\text{strain}} + E_{\text{aniso}}; D = J = \Theta/5$	T. D.	Fig. S1
Anisotropic Heisenberg	$E_{\text{MC}} = E_{\text{dip}} + E_{\text{strain}} + E_{\text{aniso}}; D = J = \Theta/10$	T. D.	Fig. S1

Table S1: Summary of MC simulations carried out as part of this study. The abbreviations 'G. S.' and 'T. D.' denote ground-state determination and temperature dependence MC simulation types, respectively.

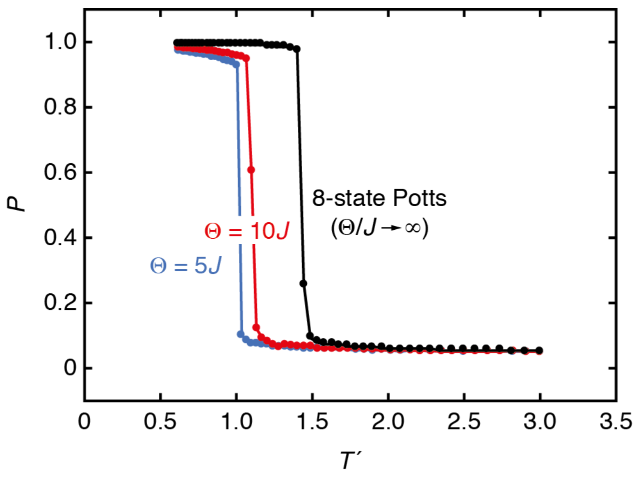

Figure S1: Temperature-dependent polarisation for the anisotropic Heisenberg MC models with strain and dipole–dipole interactions. The 8-state Potts trace is that shown in Fig. 4(a) of the main text. Evident here is that the same paraelectric/ferroelectric transition is observed for Heisenberg models with single-ion anisotropies $\Theta = 5, 10J$, albeit with a lower transition temperature. Error bars are smaller than the symbols.

2 Dipole–dipole and strain calculations

Coarse-grained ground state energies for Models 1–5 [Eq. (4) of the main text] were calculated using Eqs. (2) and (3). The analytical forms of the corresponding energies and their contributions from dipole–dipole and strain terms are collectively summarised in Table S2. Included in this table are the (fitted) coarse-grained and DFT energies used to construct Fig. 5(b) of the main text.

Model	E_{dip}	E_{strain}	E_{rel}	E_{calc} (K)	E_{DFT} (K)
**1**	$-2.094D$	$-3J$	0	0	0
**2**	0	$-3J$	$2.094D$	1744	1624
**3**	$-2.204D$	$-2.111J$	$-0.110D + 0.889J$	719	719
**4**	0	$-3J$	$2.094D$	1744	1494
**5**	0	$-3J$	$2.094D$	1744	2113

Table S2: Analytical coarse-grained energies for each of the models 1–5 discussed in the main text.

3 Density functional theory calculations

Methodology

The DFT calculations were performed using the Vienna Ab Initio Simulation Package (VASP)[S9–12]. We employed the optB86b-vdW exchange correlation potential[S6] which includes VdW corrections previously found to suit hybrid perovskites[S7]. Projector augmented-wave (PAW) pseudopotentials[S8, S9] were utilised, as supplied within the VASP package, with the following number of valence electrons treated explicitly: 9 for Rb ($4s^2 4p^6 5s^1$), 7 for I ($5s^2 5p^5$), 4 for C ($2s^2 2p^2$), 5 for N ($2s^2 2p^3$), and 1 for H ($1s^1$). We used a plane wave basis set with a 800 eV energy cutoff and a $3 \times 3 \times 3$ Monkhorst-Pack k-point mesh for the $R3$ structure (scaled accordingly for other supercells). Structures have been relaxed until the forces on any ion were less than 5 meV/Å.

Relaxed structures

The crystallographic details associated with the DFT-relaxed structures for Models 1–5 are given in Tables S3–S7. The atom labels used for MDABCO molecules are shown in Fig. S2. Atoms related to one another in the C_{3v}-symmetric MDABCO molecule but no longer symmetry-related in the corresponding ABX_3 polymorph are denoted by appending a suffix of the form ‘a’, ‘b’, or ‘c’.

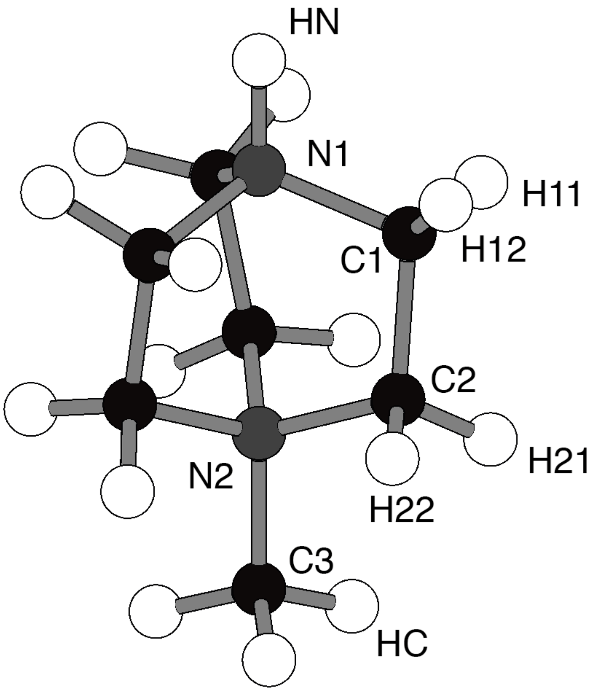

Figure S2: Atom labels used for the MDABCO molecule.

Crystal class	Rhombohedral		
Space group	$R3$		
a (Å)	7.196		
α (°)	84.52		
V (Å^3)	367.82		
Z	1		
E_{rel}/Z (meV)	0		
Atom	x	y	z
Rb1	0.9965	0.9965	0.9965
I1	0.0073	0.9345	0.5028
N1	0.6222	0.6222	0.6222
N2	0.4365	0.4365	0.4365
C1	0.7497	0.4674	0.5390
C2	0.6273	0.3346	0.4626
C3	0.3269	0.3269	0.3269
HN	0.6992	0.6992	0.6992
H11	0.8312	0.3962	0.6509
H12	0.8438	0.5336	0.4276
H21	0.6051	0.2092	0.5597
H22	0.6887	0.2940	0.3248
HC	0.1878	0.4016	0.3127

Table S3: Crystallographic details for the DFT relaxed structure of Model 1.

Crystal class	Rhombohedral		
Space group	$R\bar{3}$		
a (Å)	10.777		
α (°)	53.34		
V (Å^3)	747.04		
Z	2		
E_{rel}/Z (meV)	139.93		
Atom	x	y	z
Rb1	0	0	0
Rb2	0.5	0.5	0.5
I1	0.2264	0.7273	0.2801
N1	0.3095	0.3095	0.3095
N2	0.2180	0.2180	0.2180
C1	0.4183	0.3249	0.1307
C2	0.3841	0.2361	0.0906
C3	0.1641	0.1641	0.3269
HN	0.3473	0.3473	0.3473
H11	0.5488	0.2681	0.1173
H12	0.3854	0.4589	0.0524
H21	0.3763	0.3101	−0.0333
H22	0.4788	0.1095	0.0980
HC	0.2618	0.0474	0.1447

Table S4: Crystallographic details for the DFT relaxed structure of Model 2.

Crystal class	Monoclinic						
Space group	Pc						
a (Å)	7.163						
b (Å)	7.165						
c (Å)	14.486						
β (°)	84.51						
V (Å^3)	740.00						
Z	2						
E_{rel}/Z (meV)	62.00						
Atom	x	y	z	Atom	x	y	z
Rb1	0.0046	−0.0340	0.0090	HN	0.6775	0.7373	0.3611
I1	−0.0585	0.4672	0.0114	H11a	0.3657	0.6689	0.4125
I2	0.0233	−0.0522	0.2623	H11b	0.8150	0.4405	0.3376
I3	0.5138	−0.0495	−0.0330	H11c	0.6588	0.8461	0.2114
N1	0.6100	0.6465	0.3184	H12a	0.5001	0.4575	0.4220
N2	0.4437	0.4327	0.2146	H12b	0.8464	0.5653	0.2292
C1a	0.4446	0.5577	0.3738	H12c	0.4253	0.8467	0.2706
C1b	0.7436	0.4973	0.2798	H21a	0.2031	0.5471	0.2907
C1c	0.5414	0.7584	0.2404	H21b	0.5959	0.2283	0.2816
C2a	0.6289	0.3482	0.2352	H21c	0.5852	0.5997	0.1098
C2b	0.3243	0.4612	0.3056	H22a	0.2767	0.3231	0.3314
C2c	0.4783	0.6210	0.1688	H22b	0.7026	0.3017	0.1691
C3	0.3503	0.3072	0.1512	H22c	0.3459	0.6668	0.1434
				HCa	0.4405	0.2975	0.0856
				HCb	0.2128	0.3659	0.1393
				HCc	0.3325	0.1689	0.1834

Table S5: Crystallographic details for the DFT relaxed structure of Model 3.

Crystal class	Triclinic						
Space group	$P\bar{1}$						
a (Å)	14.201						
b (Å)	7.349						
c (Å)	7.234						
α (°)	83.84						
β (°)	85.49						
γ (°)	87.19						
V (Å^3)	747.64						
Z	2						
E_{rel}/Z (meV)	128.76						
Atom	x	y	z	Atom	x	y	z
Rb1	0	0	0	HN	0.3108	0.8008	0.6595
Rb2	0.5	0	0	H11a	0.3109	0.8792	0.3365
I1	0.2543	0.0242	0.8961	H11b	0.1602	0.7023	0.7450
I2	0	0.5	0	H11c	0.4220	0.6362	0.4410
I3	0.5	0.5	0	H12a	0.1902	0.8630	0.4335
I4	0	0	0.5	H12b	0.2410	0.5157	0.8117
I5	0.5	0	0.5	H12c	0.4059	0.5259	0.6743
N1	0.2907	0.6972	0.5817	H21a	0.3021	0.6157	0.1725
N2	0.2353	0.4533	0.3999	H21b	0.1009	0.5477	0.5089
C1a	0.2568	0.7854	0.4003	H21c	0.3770	0.3526	0.3537
C1b	0.2108	0.5987	0.6931	H22a	0.1763	0.6563	0.2064
C1c	0.3716	0.5641	0.5434	H22b	0.1490	0.3453	0.6367
C2a	0.2424	0.6329	0.2770	H22c	0.3205	0.2830	0.5793
C2b	0.1654	0.4809	0.5648	HCa	0.1344	0.3430	0.2460
C2c	0.3311	0.3987	0.4708	HCb	0.2041	0.1783	0.3857
C3	0.2053	0.3064	0.2926	HCc	0.2565	0.2947	0.1720

Table S6: Crystallographic details for the DFT relaxed structure of Model 4.

Crystal class	Triclinic
Space group	$P\bar{1}$
a (Å)	7.150
b (Å)	9.848
c (Å)	10.779
α (°)	91.39
β (°)	94.91
γ (°)	92.74
V (Å^3)	755.01
Z	2
E_{rel}/Z (meV)	182.11

Atom	x	y	z	Atom	x	y	z
Rb1	0.0086	0.7443	0.2510	HN	0.3442	0.2364	0.4740
I1	0.5149	0.7280	0.2907	H11a	0.6392	0.3397	0.4687
I2	0	0	0	H11b	0.3665	0.0211	0.3795
I3	0	0.5	0.5	H11c	0.1582	0.3473	0.3188
I4	0	0.5	0	H12a	0.4789	0.4449	0.3833
I5	0	0	0.5	H12b	0.6007	0.0932	0.4203
N1	0.3919	0.2362	0.3843	H12c	0.1432	0.1693	0.2755
N2	0.5220	0.2378	0.1737	H21a	0.7794	0.2692	0.2863
C1a	0.5453	0.3462	0.3833	H21b	0.3950	0.0392	0.1616
C1b	0.4711	0.1006	0.3583	H21c	0.3479	0.4049	0.1532
C1c	0.2366	0.2619	0.2859	H22a	0.6845	0.4210	0.2237
C2a	0.6498	0.3240	0.2668	H22b	0.6433	0.0476	0.2076
C2b	0.5092	0.0942	0.2203	H22c	0.2455	0.2479	0.0850
C2c	0.3287	0.2949	0.1668	HCa	0.7364	0.2041	0.0531
C3	0.5923	0.2390	0.0472	HCb	0.4978	0.1711	−0.0147
				HCc	0.5902	0.3433	0.0136

Table S7: Crystallographic details for the DFT relaxed structure of Model 5.

4 Hydrogen bond strengths in methyldabconium perovskites

We make the point in the main text that the strongest hydrogen-bonding interaction between MDABCO cations and the surrounding anionic perovskite cage involves the tertiary ammonium cation. While hydrogen bonding of these systems has been discussed in general terms, to the best of our knowledge there is no detailed investigation of the relative strengths of different MDABCO hydrogen–cage interactions. Consequently we employed the technique used in Ref. S5 to identify hydrogen bond strengths. The basic approach taken is as follows. First, we identified each candidate C–H$\cdots$I or N–H$\cdots$I interaction on the basis of H$\cdots$I distances. In [MDABCO]$\mathrm{RbI_3}$ there are five symmetry-distinct distances of this type; they are shown in Fig. S3 and listed in Table S8. Next, for each C–H or N–H pair, we perturbed the corresponding separation by small distances $|\Delta d| < 0.2$ Å along the C/N–H vector, and determined the DFT energy change as a function of Δd. Using the harmonic approximation

$$\Delta E_{\mathrm{DFT}} \simeq \frac{1}{2}k(\Delta d)^2, \tag{5}$$

we could extract from our data an effective force constant k for each candidate hydrogen bond interaction. We carried out the equivalent calculation for the free MDABCO cation, and then determined the hydrogen bond index Φ as follows:

$$\Phi = 1 - \sqrt{\frac{k_{\mathrm{ABX_3}}}{k_{\mathrm{free}}}}, \tag{6}$$

where $k_{\mathrm{ABX_3}}$ is the effective force constant in [MDABCO]$\mathrm{RbI_3}$ and k_{free} is that in the free cation. The larger the value of Φ, the stronger the corresponding hydrogen-bonding interaction.

Our data are given in Table S8, from which it is clear that the ammonium N–H. . .I interaction is the strongest hydrogen bond (despite the existence of shorter hydrogen bond lengths within the crystal).

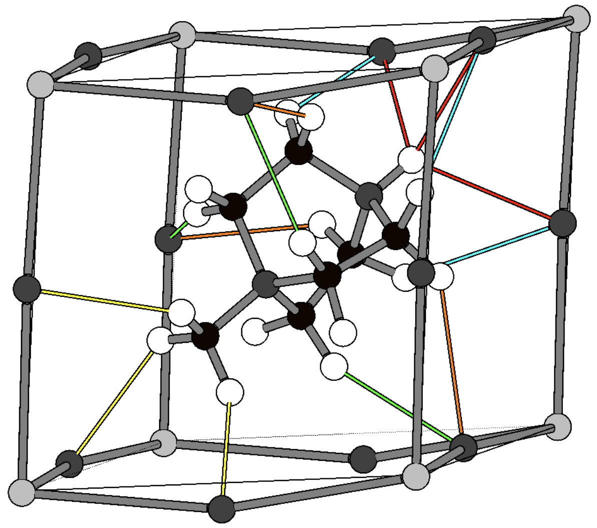

Figure S3: Close hydrogen–iodine interactions in [MDABCO]RbI_3; symmetry-related interactions are shaded in the same colour.

Hydrogen bond	Colour in Fig. S3	d(H–I) (Å)	k_{ABX_3} (meV/Å^2)	k_{free} (meV/Å^2)	Φ (%)
N1–HN· · · I	red	3.07	9.02	10.15	5.72
C1–H11· · · I	blue	3.06	9.24	9.47	1.23
C1–H12· · · I	orange	2.93	9.33	9.51	0.94
C2–H21· · · I	green	3.00	9.19	9.46	1.44
C3–HC1· · · I	yellow	2.98	9.44	9.60	0.80

Table S8: Summary of candidate hydrogen bonding interactions in [MDABCO]RbI_3. Included are the equilibrium distances d, bound and free effective force constants, and hydrogen bond index Φ.

5 Symmetry implications of (anti)ferroelastic distortions

In the main text we discuss the implications of ferroelastic and antiferroelastic strains for distortions of the B-site coordination environment. We elaborate on the point here by making clear our symmetry arguments.

In the polar ferroelastic $R3$ phase, the B-site cation is located on the $3a$ Wyckoff site, with 3. point symmetry. The corresponding C_3 axis lies normal to one pair of faces of the RbI_3 octahedron. Hence the $R3$ state allows any distortion of the RbI_3 coordination environment that preserves this three-fold symmetry. This includes, in particular, rotations and anti-rotations (distortions towards a trigonal prismatic geometry) of the polyhedron that give bending both of Rb–I–Rb and I–Rb–I bond angles.

By contrast, the competing antiferroelastic phase has $I23$ space group symmetry, and the B-site cations are located on the $2a$ and $6b$ Wyckoff positions. These have 23. and 222.. point symmetry, respectively. In both cases, two-fold rotation axes pass through each of the Rb–I bond vectors, which constrains the corresponding Rb–I–Rb angles to be $180°$ and the I–Rb–I angles to be $90°$. Hence there are no symmetry-allowed polyhedral rotations or bending modes in this state. We anticipate this raises the energy of the antiferroelastic phase, which is why the strain coupling strength J is positive, rather than negative, for [MDABCO]RbI_3.

6 References

(S1) J. A. M. Paddison, H. Jacobsen, O. A. Petrenko, M. T. Fernández-Díaz, P. P. Deen, and A. L. Goodwin, *Science* **350**, 179 (2015).

(S2) Z. Wang and C. Holm, *J. Chem. Phys.* **115**, 6351 (2001).

(S3) Z. Wang, C. Holm, and H. W. Müller, *J. Chem. Phys.* **119**, 379 (2003).

(S4) S. W. de Leeuw, J. W. Perram, and E. R. A. Smith, *Proc. R. Soc. London A* **373**, 57 (1980).

(S5) X.-Z. Li, B. Walker, and A. Michaelides, *Proc. Natl. Acad. Sci., U.S.A.* **108**, 6369 (2011)

(S6) J. Klimeš, D. R. Bowler, and A. Michaelides, *Phys. Rev. B* **83**, 195131 (2011)

(S7) J.-H. Lee, N. C. Bristowe, J. H. Lee, S.-H. Lee, P. D. Bristowe, A. K. Cheetham, and H. M. Jang, *Chem. Mater.* **28**, 4259 (2016).

(S8) P. E. Blöchl, *Phys. Rev. B* **50**, 17953 (1994)

(S9) G. Kresse and J. Furthmüller, *Phys. Rev. B* **54**, 11169 (1996)

(S10) G. Kresse and J. Hafner, *Phys. Rev. B* **47**, 558(R) (1993)

(S11) G. Kresse and J. Furthmüller, *Comput. Mater. Sci.* **6**, 15 (1996)

(S12) G. Kresse and J. Hafner, *Phys. Rev. B* **49**, 14251 (1994)
